# Cholera—By Prof. Alonzo Clark, M. D.

**Published:** 1866

**Authors:** 


### Miscellaneous.

### Cholera---By Prof. Alonzo Clark, M. D.

*Being a full synopsis of Lectures on Cholera, recently delivered at the College of Physicians and Surgeons, New York, and specially reported for the Medical and Surgical Reporter.*

### CAUSES AND NATURE OF CHOLERA.

The next point in the consideration of this disease is its cause, and the mode in which that cause acts upon the system, so as to produce the various phenomena, which have already been noticed.

The literature of the subject abounds with a variety of theories of the manner in which the cause of cholera acts. One theory is that the disease is zymotic in a certain degree, in other words, that after the introduction of the poison into the system, by a process of fermentation of some sort, a poison is produced like that which was at first introduced, if not in the body of the patient himself, yet in the excretions.

Another very prevalent theory is that there exists during a cholera epidemic, and as its cause, a peculiar condition, the so-called *epidemic constitution* of the atmosphere, which is widely diffused, and acting as the general cause, brings on an outbreak of the disease whenever it meets certain local circumstances and causes, the so-called *localizing conditions.*

Dr. Snow has another theory, according to which the disease is a communicable one in a particular way; he claims that in the alimentary canal of the cholera-patient a poison is produced, which consists of a something capable of being absorbed by or floating in water; that the water of wells and cisterns, during an epidemic, becomes impregnated by this poison and causes the spread of the disease; that a reason why the disease is so much more frequeutly communicated to nurses and attendants around the patient than to physicians, is because the former are less cleanly, and hence more frequently subject to introduction of this peculiar poison into the system.

In regard to this theory, certain things must be taken in consideration. Dr. Snow claims that the poison is generated in the alimentary canal, and carried by the evacuations and discharges from the stomach and bowels. Now, it is testified to by Schmidt, of Münich, that a man, in a state of intoxication, drank a large beer-glassful of the vomit of a cholera patient, without being followed by the first symptom of cholera, and physicians of Münich are said to have freely tasted and even swallowed the choleraic transudations, without ill effects.

Again, a noticable fact in the geographical course of cholera, is that it almost uniformly ascends rivers and streams; thus it ascended the Volga, Thames, Hudson, and the Mississippi. If the disease was propagated by the water contaminated by cholera dejections and evacuations, it is plain that it should descend in its course. In regard to some rivers it may be objected that they are not resorted to for drinking and domestic purposes. But in others which are thus freely used, as for instance the Mississippi, the disease has always traveled up-stream, not down.

Another theory, which indeed may be looked upon as but a modification of the former, is that adopted by several German physicians, which also considers a peculiar poison to result from the discharges of the patients, but not immediately. The cholera discharges, according to Thiersch, are not poisonous at first, but become so, after the lapse of some time, by decomposition and fermentation under an elevation of temperature of at least 50° Fahrenheit. According to facts brought forward in support of this theory, this fermentation which develops the poison in the discharges ceases in about eight days, and then they become inert.

The chief evidence upon which Thiersch bases his opinion is that he fed a number of mice on fresh cholera discharges, and a number on cholera material which had undergone fermentation. The animals fed on the first remained perfectly well, while those that had been living on the fermented discharges were killed by it, under symptoms resembling cholera, diarrhœa taking place before death, and post-mortem appearances being analogous to those in man. Animals which were fed on old cholera evacuations, after fermentation had ceased, suffered as litte as the first.

The logical conclusion, if this theory be true, is that means of disinfecting the evacuations of cholera patients by chloride of lime, sulphuric acid, etc., constitute a chief and important element of prophylaxis. In support of this, it is stated that there are two prisons in the neighborhood of Münich; in one very strict and energetic measures were adopted, during the prevalence of the disease, to disinfect the discharges of all the prisoners and inmates, and the result was that only *one* case occurred among five hundred inmates; in the other institution, in which no means whatever were adopted of disinfecting the discharges, 15 per cent. of the inmates were attacked.

The evidence in regard to this theory is not yet conclusive; still it deserves attention, and should remain open for further investigation.

Another theory which has been advanced is that the influence of *ozone*, negative or positive, in the atmosphere, is connected with the causation of the disease. There are many quite opposite opinions regarding this theory, and the influence of the presence or absence of ozone in abnormal quantity. Dr. Peters, of Lexington, Ky., during the prevalence of a cholera epidemic, instituted investigations as to this point, and according to his statements, not much change in the ozonic condition of the air could be observed; if there was any change, it was unimportant. The most recent observations probably on this subject are those of Dr. B. W. Richardson, of England. Some of his conclusions as to the facts at preseut known respecting ozone are stated as follows: It is always present in the air, naturally in the proportion of about 1 part in 10,000; it is rapidly destroyed in large towns, crowded, close, and filthy localities; ozone gives to oxygen its life-supporting properties; its effects are destroyed by great heat; diffused minutely through the air, it produces, on inhalation, distinct symptoms of acute catarrh. When animals are subjected for a long period to ozone in small proportions, the agent acts differently in different animals; carnivora die, after some hours, from disorganization of the blood, while herbivora will live for weeks and suffer from no acute disease. Science has not yet actually *demonstrated* that the presence of ozone in the air can produce actual disease, though the facts approach to demonstration that catarrh is thus produced. During periods of intense heat of weather, the ozone loses its active power. Ozone acts rapidly upon putrefactive organic matter, breaking up the ammoniacal products of decomposition, and hastening organic destruction. In a negative condition of air, i. e.

the absence of ozone, the purification of the organic matter is greatly modified, and the offensive products are increased; wounds become unhealthy and heal slowly in such negative air, and though there is no demonstrative evidence that any diseases are actually caused by this negative condition, the inference is fair that diseases which show a putrefactive tendency are influenced injuriously by a negative condition of the oxygen of the air. It is also probable that during this state, decomposing organic poisonous matters become more injurious. And finally, as ozone is used up in crowded localities, and as it is essential that ozone should be constantly supplied, in order to sustain the removal of decomposing substances and their products, no mere attention to ventilation and other measures can be fully effective, unless the air introduced be made active by ozone. Fever hospitals and other large buildings in towns should be artificially fed with ozonized air.

All, however, that is positively known regarding ozone, in the present state of science, is a bare probability that if it exists in certain quantities, it may purify the air. As to its casual connection with cholera, nothing is positively known.

*Electricity*, too, has been charged with being the cause of cholera; at least it has been claimed that its presence or absence has something to do with its production. A French observer has made a statement that he observed, during the prevalence of cholera, that he could not obtain sparks as usual from an electrical apparatus, and in England it is said to have been noticed that, during a cholera epidemic, a large horse-shoe magnet attracted and held suspended much larger quantities of iron than ordinarily, and that as cholera abated and ceased, its attractive power diminished also.

These and similar observations, however, do not bear sufficient weight to justify any decided conclusions.

Leaving the question as to the influence of ozone, still another theory has been proposed by Dr. Mitchell, who finds the cause of cholera to be a *fungus* or fungoid spores, developed in the earth or in the atmosphere, and introduced into the system by the respiration. In another branch of this theory, the origin and growth of the fungus is placed into the body itself. This theory received a special importance when Drs. Brittain and Swain announced that they had found in the drinking water of London the *cholera fungi*, which were minutely described. At Edinburgh, however, these fungi could not be detected with the microscope, neither in the drinking water nor in the dejections of the patients. Dr. Parker, the microscopical anatomist of the London Hospital, states that fungi of various sorts are not unfrequently found in the intestinal canal of animals and man, but that they are perfectly inert. The strongest reason, however, for discrediting this statement is in the result of the investigations of a committee appointed by the College of Physicians of England, who examined into the matter. They reported that the *rings*, which were described as forming part of the cholera fungi by Swain, were found to be remnants of onions, turnips, and other vegetables; the *oval cells*, also claimed as part of the cholera fungi, were ordinary spores of the rust of wheat or corn, such as are frequently found in bread; and the *disk*, also part of the supposed cholera fungus, had a similar origin. So, on the whole, this idea comes to nought, as well as Dr. Snow's theory; unless further and stronger evidence is produced.

Another view is, that it is produced in a manner similar to yellow fever. This theory claims that there are certain wide-spread conditions, atmospheric or terrestrial, which produce a certain predisposition; and that added to these a special miasm is produced, external to ourselves; a certain poison is liberated in the air, unknown in its nature, intangible, known only by its effects, which will re-produce itself, and so render a whole town infected. This we know of yellow fever, which is not an indigenous disease, but can be kept off by rigid quarantine and strict sanitary rule. In just this way it seems cholera is produced—by a special poison, a malaria, the production of certain general wide-spread conditions and local influences, but not re-produced in the system.

All these various theories agree in one point, namely, that *there is a poison* which gives rise to the disease, and this is a fact which cannot be disbelieved.

How this poison is introduced into the body cannot be confidently answered. We might assume that it is absorbed through the surface of the skin, the lungs, or the alimentary canal, etc. But, in one way or another, it is introduced, enters the blood, and circulates through the system.

Introduced into the system, the poison of cholera soon begins to act upon *the ganglionic nervous system.* It is true, the examinations made after death, of the centres and nerves of the ganglionic system have not led to constant appearances; and, indeed, they are not found to have undergone marked changes, except occasional softening and enlargement. But still this is the best hypothesis which we can present, as to the manner in which the poison of cholera acts upon the system, and that we can find no decided anatomical lesions, is no real excuse for not accepting it. A thousand changes are produced and observed in the innervation of the organs and tissues of the body, characterized by the most marked phenomena, without our being able to discover any physical appearances in the nerve structure, which would account for them. Certain medicinal substances appear to have specific affinities in their action, to different parts of the nervous system; strychnia, for instance, upon the spinal system, opium upon the brain. Now, in a case of strychnia-poisoning you observe the most fearful commotion of all the muscles controlled by the spinal axis, resulting in death. Still, if you search after death for any marked anatomical lesions in the spinal cord or nerves, none will be found. Thus, again in a person dead from an over-dose of opium, though congestion may be found in the vessels and meninges of the brain, the most careful examination will not discover any microscopical changes in the brain-cells or the minute structure of the organ. Thus certain agents, indisputably, exert a powerful action upon various parts of the nervous system, with no accompanying special lesion that can be observed; we might run through the whole of the medicinal articles acting upon the nerves to illustrate this point.

While, then, the absence of pathological evidences after death forms no objection to this hypothesis, there are certain analogies which speak strongly in its favor. The branches or filaments of the sympathetic nerve, which go to the eye, govern and regulate the circulation of that organ; if they are divided or disordered, you observe hyperæmia, echymosis, and a series of changes, which finally lead to ulceration and destruction of the organ. So the ganglionic system everywhere, but more particularly in the alimentary canal, governs and regulates capillary circulation. The poison of cholera begins to act upon the ganglionic system, partially paralyzing it, and gradually increasing in its effects during the period of diarrhœa. The blood flows into these vessels in unusual tides, giving rise to hyperæmia more or less intense, and from which the copious discharges are derived; this goes on until the blood, by the constant drain of its fluid constituents becomes markedly and seriously changed; then follows the disturbance in the spinal system, the cramps and convulsions, which, it is probable, are entirely reflex. The hyperæmia at first does not manifest a tendency which it afterwards shows; but, as it continues, much as in the eye after division of the branches of the sympathetic nerve, assumes an inflammatory character, for we cannot, on looking at the post-mortem appearances avoid the conclusion that there is inflammatory hyperæmia, or even a certain degree of inflammation. There is, however, no organizable, plastic deposit. The material thrown out, as described in a former lecture, is something like the exudation of diphtheria; and, it may be added here, that this infiltration of granular matter into the intestinal mucous membrane and glands, seems to be almost characteristic of the disease.

Dr. Horner, as early as 1832, noticed and described the detachment and denution of epithelium, and sloughing of portions of the mucous membrane; and he believed the disease to be inflammatory. Pirogoff, too, says that the cholera appearances have great analogy to inflammation.

In summing up the evidence regarding the cause and nature of cholera, it may be stated then, that there is a poison, the exact nature of which is not yet perfectly understood; that this poison, introduced into the system, causes disturbances of innervation, or a sort of paralysis of the ganglionic nervous system; that this leads to extensive hyperæmia of the alimentary canal, resulting in the symptoms described, and to the reflex phenomena alluded to, and as the disease progresses, obtaining more or less an inflammatory character.

